# Dr. Brown, on Ascarides

**Published:** 1802-03

**Authors:** Joseph Brown

**Affiliations:** Queen's Lane, Lower Street, Islington


### *To the Editors of the Medical and Phyfical Journal.*

GENTLEMEN,

I Am one among a great number of the Readers of your excellent Medical Mifcellany, who are doubtlefs very much obliged, and highly entertained, by the hints that from time to time we meet with in the Phyfical Journal, concerning the medical practice and ufeful difcoveries of foreign as well as of domeftic practitioners in the noble art of healing. But altho', in general, I wait with fome degree of impatience for the monthly appearance of the communications of your ingenious and inftructive Correfpondents, together with the refult of your learned lucubrations, it was not till this moment that I obferved it it announced (see page 378 of your laſt volume,) as a practice peculiar to Prof. HUFELAND, to employ clyſters of lime-water, to free a patient from thoſe ſmall troubleſome white worms called aſcarides, a ſpecies the moſt frequent and dangerous in this country, and which ſometimes produce the moſt alarming and fatal ſymptoms.

Happy as we ought always to be, to recognize the merit of ingenious and communicative foreigners, I cannot forbear wiſhing that your readers may be informed that Profeſſor Hufeland's method of removing aſcarides, is neither "*new nor rare*;" for, more than ten years ago, I had the honour to hear that ſkilful and eloquent teacher, Mr. CLINE, when delivering Lectures on Anatomy at St. Thomas's Hoſpital, recommend the ſimple but efficacious remedy above mentioned, to be employed in the ſame manner, and for the ſame intentions.

In two inſtances that fell under my own obſervations, when clyſters of lime-water alone did not appear to be ſo effectual, in every other inſtance in which I had an opportunity to employ them, I directed a ſmall proportion of *tinctura aloes compoſita* to be added to the *aqua calcis*; in the other caſe, complete ſucceſs attended the uſe of clyſters of lime-water prepared with a decoction of the bitter herbs or ſhrubs, as *abſinthium vulgare*, *Abrotonum*, *et Matricaria*.

Strong lime-water thus prepared, (or the herbs may be added to the lime previouſly to pouring the boiling water upon it,) when thrown over a garden by a watering pot, is one of the beſt preventives of the ravages that are often occaſioned by ſlugs and caterpillars. Garden ſlugs are alſo killed by urine that *has been kept two or three days*; but that cannot be ſafely thrown near the roots of plants or trees, nor upon them, though it is extremely uſeful to prevent the breeding of vermin, when it is thrown on dung-hills with which the ground is to be manured, but lime-water prepared with a ſtrong decoction, or by infuſing in it the bitter plants, or ſhrubs, will, without injury to vegetation, be found eminently ſerviceable for deſtroying thoſe rapacious inſects.

The lovers of Medical Botany, and thoſe who have a taſte for the pure pleaſures of Horticulture, will, I truſt, with yourſelves, Gentlemen, excuſe this digreſſion of

Your's, &c.

JOSEPH BROWN, M. D.

*Queen's Lane, Lower Street, Islington, Dec.* 10, 1801.

